# Determinants of unmet need for family planning in Gambia & Mozambique: implications for women’s health

**DOI:** 10.1186/s12905-021-01267-8

**Published:** 2021-03-23

**Authors:** Sanni Yaya, Dina Idriss-Wheeler, Olalekan A. Uthman, Ghose Bishwajit

**Affiliations:** 1 School of International Development and Global Studies, Faculty of Social Sciences, University of Ottawa, 120 University Private, Ottawa, ON K1N 6N5 Canada; 2 The George Institute for Global Health, Imperial College London, London, UK; 3 Faculty of Health Sciences, University of Ottawa, Ottawa, Canada; 4 Warwick Centre for Applied Health Research and Delivery (WCAHRD), Division of Health Sciences, Warwick Medical School, University of Warwick, Coventry, CV4 7AL UK

**Keywords:** Family planning, Gambia, Mozambique, Unmet need, Women’s health

## Abstract

**Background:**

In low-middle-income countries, unmet need for family planning (FP) constitutes a major challenge for prevention of unintended pregnancies and associated health and psychological morbidities for women. The factors associated with unmet need for family planning have been studied for several countries in sub-Saharan Africa, but not much is known about the situation in Gambia and Mozambique. The purpose of this study was to perform a comparative analysis of the prevalence of unmet need for FP, and its sociodemographic correlates in Gambia and Mozambique to better inform FP policies and programs aimed at reducing associated negative health outcomes for women and their families.

**Methods:**

In this analysis we used nationally representative data from Demographic and Health Surveys in Gambia (2013) and Mozambique (2011). Sample population were 23,978 women (n = 10,037 for Gambia and 13,745 for Mozambique) aged 15–49 years. Women who want to stop or delay childbearing but were not using any contraceptive method were considered to have unmet need for FP. Association between unmet need for FP and the explanatory variables was measured using binary logistic regression models

**Results:**

Prevalence of unmet need for FP was 17.86% and 20.79% for Gambia and Mozambique, respectively. Having employment in professional/technical/managerial position showed an inverse association with unmet need both in Gambia [OR = 0.843, 95% CI 0.730, 0.974] and Mozambique [OR = 0.886, 95% CI 0.786, 0.999]. Education and household wealth level did not show any significant association with unmet need. The only positive association was observed for rural [OR = 1.213, 95% CI 1.022, 1.441] women in the richer households in Gambia. Having access to electronic media [OR = 0.698, 95% CI 0.582, 0.835] showed a negative effect on having unmet need in Mozambique. Women from female headed households in Gambia [OR = 0.780, 95% CI 0.617, 0.986] and Mozambique [OR = 0.865, 95% CI 0.768, 0.973] had lower odds of unmet need for FP.

**Conclusion:**

The situation of unmet need for FP in Gambia and Mozambique was better than the Sub-Saharan African average (25%). Nonetheless, there is room for improvement in both countries. Significant assocations with lower unmet need for family planning and women’s occupational status (more education & higher skilled employment), access to mass media communication, and female-headed households provide possible areas for intervention for improved FP opportunities in the region.

**Supplementary Information:**

The online version contains supplementary material available at 10.1186/s12905-021-01267-8.

## Background

Unmet need for family planning is defined as the percentage of women of reproductive age, either married or in a union, who have an unmet need for family planning [1]. Family planning refers to the services, policies, information, attitudes, practices, and commodities, including contraceptives, that give women, men, couples, and adolescents the ability to avoid unintended pregnancy and choose whether and/or when to have a child [2]. The definition is restricted to individuals of reproductive age who are either married or in a union, and is generally categorized into two levels: those who have no desire to have more children at all, and those who desire to have children in future but want to delay conception for a certain period of time. The concept of unmet need for FP has been a popular topic in population health research and policy decision-making for over three decades [3]. Although unmeet for FP is a global issue, it appears to be more concentrated in the low-middle-income countries (LMICs) which tend to be characterized by higher fertility and maternal and child mortality rates.

In sub-Saharan Africa, where fertility remains the highest in the world, considerable efforts have been made by national and international actors to address the low coverage and usage of modern contraceptive techniques [4, 5]. On average, the African continent has achieved appreciable progress in terms of promoting the quality of reproductive healthcare and reducing the burden of maternal and child mortality. Nonetheless, the goals remain elusive for certain countries which are striving to meet the Millennium (MDG 5b) and Sustainable Development Goals (SDGs 3.7 and 5.6) [2]. Between 1990 and 2010, the global prevalence of unmet need for FP decreased from 15.4 to 12.3% [6]. However, the progress has been unequal across regions with sub-Saharan Africa having the lowest level of contraceptive prevalence rate (24% as well as the highest level of unmet need (25%) [7], and of unintended pregnancy (29%) [8].

Several studies have been conducted on FP issues in Gambia [9, 10] and Mozambique [5, 11]. These studies focused on the use of contraceptive methods from supply and demand perspectives and the barrier to accessing FP services. To date, there are no studies on unmet need for FP and associated factors in either country, especially using a nationally-representative sample. Both Gambia and Mozambique have higher than average rates of poverty (per capita GDP reported at $518 US and $415 respectively in 2018), as well as maternal (597 and 289 deaths/100,000 live births respectively in 2017) and child (58.4 and 73.2 deaths/1000 live births respectively in 2018) mortality rates. Furthermore, both countries have high HIV rates, especially Mozambique which has the 8th highest HIV rate in the world [12].

Widespread poverty and high unmet need for FP are two important risk factors that can exacerbate the HIV epidemic [13–16]. Hence, it is believed that addressing unmet need for FP will not only contribute to better maternal and child health outcomes, but also to better control prevalence of HIV-infected individuals and mother-to-child HIV transmission [13]. The factors that lead to unmet need can stem from various demographic, sociocultural, and environmental causes. Understanding the associated factors is central for designing effective FP policies and programs aimed at reducing or eliminating those barriers. In this study, the authors aimed to enhance the understanding of the factors of unmet need of FP in Gambia and Mozambique using data from two Demographic and Health Surveys. The prevalence and contextual factors associated with unmet need for FP were assessed to answer the research question: what are the prevalence and predictors of unmet need for contraception in Gambia and Mozambique? Such evidence can help reinforce the efforts to combat the adverse outcomes of unmet need for FP including unplanned pregnancies and unsafe abortions.

## Methods

### Data source

Data for this study were collected from Gambia (2013) and Mozambique (2011) Demographic and Health Survey (DHS). In Gambia, the survey was implemented by Gambia Bureau of Statistics and the Ministry of Health and Social Welfare; and in Mozambique by the National Statistical Institute (Instituto Nacional de Estatística) and Ministry of Health (MISAU). Technical assistance for DHS is provided by Inner City Fund (ICF) International and financial support from both governments, the U.S. Agency for International Development (USAID), the United Nations Population Fund (UNFPA), the United Nations Development Programme (UNDP), the United Nations Children’s Fund (UNICEF), the Joint United Nations Programme on HIV/AIDS (UNAIDS), the World Health Organization (WHO), and the Global Fund. Data collection in Gambia took place from February to April 2013 and June to November 2011 in Mozambique. The complete DHS sample population includes men (15–54 years), women (15–49 years) and children (< 5 years). However, for this study, only the women’s sample was used which included interviews completed by 10,037 women of reproductive age in Gambia and 13,745 women of reproductive age in Mozambique.

### Definition of the variables

The answer to the question on unmet need included the following options: never had sex; has unmet need for spacing; has unmet need for limiting; using contraception for spacing; using for limiting; no unmet need; not married and no sex in last 30 days; infecund, menopausal. Women with unmet need for FP are those who are fecund and sexually active but are not using any method of contraception, and report not wanting any more children or wanting to delay the next child. For this study, only those observations for which unmet need for contraception was applicable were kept (e.g. not infecund). The respondents were categorized as (1) no unmet need (currently using any method for spacing and limiting) and (2) unmet need (not using any method) [17].

### Explanatory variables

A list of explanatory variables was made based on literature review and availability of the variables in the dataset: Age (15–19/20–24/25–29/30–34/35–39/40–44/45–49); Residency (Urban/Rural); Education (No Education/Primary/Secondary/Higher); Occupation (Not Working/Professional & Technical & Managerial/Agricultural & Self Employed); Household wealth quintile (Poorest/Poorer/Middle/Richer/Richest); has Electronic media access (No/Yes); Religion (Islam Other); Sex of household head (Male/Female); Ever had a terminated pregnancy (No/Yes). Household wealth quintile is a measure of financial well-being that is calculated based on the possession of durable goods (building material, car) by the households. The items possessed serve as the basis for ranking the households on a scale and split into five equal percentiles, with the highest quintile representing the richest households and so on [18]. Electronic media access was assessed by the use of TV and radio.

### Data analysis

Data were analyzed with Stata version 14. As the surveys used cluster sampling techniques, all analyses were adjusted for this by using the *svy* command [19]. This command uses the information on sampling weight, strata, and primary sampling unit provided with the datasets. Sample characteristics were described as percentages. Prevalence of unmet need for FP was shown as percentages across the explanatory variables separately for two countries. Association between unmet need for FP and the explanatory variables was measured using binary logistic regression models. Results of three outcome variables were presented in separate tables, each divided into three subsamples: overall, urban and rural. The results were presented as odds ratios with 95% confidence intervals (CIs). Level of significance was set at alpha value of 5%. Following the analysis, multi-collinearity among the variables using the post-estimation command of variance inflation factor (VIF) was completed. No multi-collinearity was detected as VIF values were below 10 for all the models. An additional file for the research methodology flowchart provides further details [see Additional file 1].

## Results

Sociodemographic characteristics of the sample population were summarized in Table 1. Prevalence of unmet need for FP was 17.86% and 20.79% for Gambia and Mozambique, respectively. The prevalence was relatively higher among women aged 20–29 years, in rural areas, had no/primary education, had no employment, had access to electronic media, followers of Islam, lived in male headed households, and never had any terminated pregnancy. Household wealth status did not show any noticeable difference in terms of having unmet need for FP.

**Table 1:** Sociodemographic profile of women of reproductive age who reported on unmet need for Family Planning in Gambia and Mozambique

	Gambia			Mozambique		
	N	No unmet need (82.14%)	Has unmet need (17.86%)	N	No unmet need (79.21%)	Has unmet need (20.79%)
Age						
15–19	2454	27.05 [25.90, 28.23]	6.76 [5.49, 8.30]	3065	23.04 [22.10, 24.01]	19.31 [17.65, 21.08]
20–24	2087	21.31 [20.17, 22.50]	17.93 [15.86, 20.21]	2468	17.6 [16.70, 18.53]	18.84 [17.23, 20.57]
25–29	1750	16.38 [15.31, 17.50]	24.9 [22.42, 27.56]	2340	16.13 [15.31, 16.98]	18.17 [16.59, 19.87]
30–34	1471	13.64 [12.79, 14.55]	19.73 [17.54, 22.11]	1975	14.26 [13.49, 15.06]	15.59 [14.10, 17.20]
35–39	1090	9.38 [8.59, 10.23]	14.61 [12.85, 16.56]	1691	11.8 [11.08, 12.57]	14.44 [13.05, 15.95]
40–44	761	6.76 [5.95, 7.68]	10.67 [8.82, 12.85]	1156	8.37 [7.75, 9.04]	8.66 [7.50, 9.97]
45–49	569	5.47 [4.86, 6.15]	5.41 [4.29, 6.80]	1050	8.8 [8.15, 9.50]	5 [4.12, 6.04]
Residency						
Urban	4487	57.56 [51.64, 63.28]	49.08 [42.89, 55.30]	5804	34.62 [31.95, 37.39]	35.14 [31.64, 38.81]
Rural	5695	42.44 [36.72, 48.36]	50.92 [44.70, 57.11]	7941	65.38 [62.61, 68.05]	64.86 [61.19, 68.36]
Education						
No education	5040	43.8 [41.09, 46.55]	58.67 [54.83, 62.41]	3773	31.66 [29.65, 33.74]	29.59 [26.85, 32.50]
Primary	1434	13.38 [12.23, 14.63]	15.43 [13.25, 17.89]	6774	49.66 [47.88, 51.43]	52.47 [49.77, 55.17]
Secondary	3260	36.82 [34.50, 39.21]	22.86 [20.13, 25.85]	2943	17.24 [15.69, 18.92]	16.96 [14.79, 19.36]
Higher	448	6.0 [4.95, 7.25]	3.04 [2.06, 4.46]	255	1.44 [1.02, 2.03]	0.98 [0.62, 1.55]
Occupation						
Not working	4939	51.76 [49.18, 54.33]	43.3 [39.44, 47.23]	7448	53.72 [51.79, 55.63]	54.05 [50.95, 57.13]
Professional/technical/managerial	2403	26.91 [24.46, 29.51]	28.46 [25.11, 32.06]	2645	15.78 [14.65, 16.98]	15.77 [14.17, 17.51]
Agricultural—self Employed	2780	21.33 [17.96, 25.14]	28.25 [23.99, 32.93]	3652	30.5 [28.34, 32.76]	30.18 [26.76, 33.83]
Wealth index						
Poorest	2131	16.61 [14.05, 19.53]	18.93 [15.95, 22.33]	1833	19.04 [17.04, 21.21]	18.34 [15.39, 21.71]
Poorer	2238	17.64 [15.45, 20.07]	21.76 [18.58, 25.32]	2109	18.57 [17.16, 20.06]	18.52 [16.46, 20.77]
Middle	1979	18.46 [16.30, 20.83]	20.6 [17.98, 23.49]	2399	18.9 [17.35, 20.56]	18.1 [16.13, 20.25]
Richer	1703	21.08 [18.52, 23.89]	19.84 [16.49, 23.67]	2946	19.91 [18.19, 21.74]	21.54 [18.99, 24.34]
Richest	2131	26.21 [22.73, 30.00]	18.86 [15.46, 22.82]	4458	23.59 [21.42, 25.91]	23.5 [20.88, 26.33]
Media access						
No	1008	7.62 [6.18, 9.36]	10.46 [8.12, 13.37]	3629	28.17 [26.50, 29.91]	26.05 [23.94, 28.27]
Yes	9100	92.38 [90.64, 93.82]	89.54 [86.63, 91.88]	10116	71.83 [70.09, 73.50]	73.95 [71.73, 76.06]
Religion						
Islam	9866	95.61 [93.26, 97.16]	96.41 [93.16, 98.15]	9997	70.25 [67.77, 72.62]	72.59 [69.48, 75.49]
Other	304	4.39 [2.84, 6.74]	3.59 [1.85, 6.84]	3748	29.75 [27.38, 32.23]	27.41 [24.51, 30.52]
Sex of household head						
Male	7846	75.73 [73.00, 78.28]	82.65 [79.40, 85.49]	8501	64.12 [62.52, 65.69]	66.75 [64.40, 69.01]
Female	2336	24.27 [21.72, 27.00]	17.35 [14.51, 20.60]	5244	35.88 [34.31, 37.48]	33.25 [30.99, 35.60]
Ever had a terminated pregnancy						
No	9230	91.2 [90.09, 92.19]	88.35 [85.94, 90.39]	12423	90.85 [90.02, 91.61]	92.61 [91.35, 93.70]
Yes	947	8.8 [7.81, 9.91]	11.65 [9.61, 14.06]	1322	9.15 [8.39, 9.98]	7.39 [6.30, 8.65]

Table 2 summarizes the factors of association with unmet need for FP in Gambia and Mozambique. Age was not a significant predictor of unmet need in Gambia, whereas in Mozambique higher age groups showed an inverse association especially among urban women. Women aged 45–49 years in the urban [Odds ratio = 0.463, 0.333, 0.642] and rural [Odds ratio = 0.382, 95% CI 0.284, 0.514] had lower odds of reporting unmet need. Educational level did not show any association with unmet need except for rural women in Gambia who had primary education [Odds ratio = 1.377, 95% CI 1.128, 1.680].

**Table 2:** Predictors of unmet need for Family Planning in Gambia and Mozambique

	Gambia			Mozambique		
	Pooled	Urban	Rural	Pooled	Urban	Rural
Age						
15–19	1	1	1	1	1	1
20–24	1.009 [0.794, 1.283]	1.139 [0.746, 1.738]	0.936 [0.696, 1.257]	0.748*** [0.642, 0.872]	0.732** [0.584, 0.917]	0.756** [0.613, 0.934]
25–29	1.159 [0.911, 1.476]	1.104 [0.719, 1.698]	1.187 [0.883, 1.595]	0.714*** [0.608, 0.838]	0.581*** [0.455, 0.743]	0.836 [0.675, 1.035]
30–34	1.034 [0.806, 1.326]	0.901 [0.579, 1.402]	1.118 [0.823, 1.518]	0.698*** [0.590, 0.826]	0.508*** [0.388, 0.665]	0.864 [0.694, 1.076]
35–39	1.017 [0.783, 1.320]	0.898 [0.567, 1.422]	1.093 [0.792, 1.509]	0.757** [0.636, 0.900]	0.670** [0.511, 0.878]	0.831 [0.662, 1.044]
40–44	1.057 [0.800, 1.396]	0.860 [0.523, 1.415]	1.181 [0.841, 1.659]	0.671*** [0.553, 0.814]	0.546*** [0.402, 0.742]	0.778 [0.605, 1.000]
45–49	0.810 [0.596, 1.101]	0.721 [0.420, 1.235]	0.874 [0.600, 1.273]	0.414*** [0.333, 0.515]	0.463*** [0.333, 0.642]	0.382*** [0.284, 0.514]
Residence (urban)	1			1		
Rural	1.064 [0.886, 1.277]			0.986 [0.875, 1.111]		
Education (none)	1	1	1	1	1	1
Primary	1.272** [1.085, 1.493]	1.085 [0.829, 1.422]	1.377** [1.128, 1.680]	1.099 [0.987, 1.224]	1.169 [0.925, 1.477]	1.087 [0.961, 1.229]
Secondary	1.029 [0.882, 1.199]	0.917 [0.740, 1.137]	1.118 [0.894, 1.397]	1.018 [0.870, 1.192]	0.988 [0.760, 1.283]	1.214 [0.951, 1.551]
Higher	0.747 [0.520, 1.072]	0.673 [0.447, 1.012]	0.913 [0.386, 2.158]	0.808 [0.552, 1.184]	0.878 [0.569, 1.354]	0.511 [0.0607, 4.307]
Occupation (none)	1	1	1	1	1	1
Professional/technical/managerial	0.843* [0.730, 0.974]	0.818* [0.676, 0.990]	0.876 [0.698, 1.099]	0.886* [0.786, 0.999]	0.850* [0.729, 0.991]	0.985 [0.806, 1.203]
Agricultural—self employed	0.891 [0.777, 1.021]	0.715 [0.494, 1.035]	0.939 [0.805, 1.097]	0.887* [0.799, 0.986]	0.863 [0.687, 1.083]	0.911 [0.808, 1.028]
Wealth quintile (Poorest)	1	1	1	1	1	1
Poorer	1.134 [0.970, 1.325]	1.112 [0.632, 1.957]	1.128 [0.958, 1.327]	1.083 [0.923, 1.270]	1.472 [0.863, 2.512]	1.060 [0.897, 1.254]
Middle	1.063 [0.901, 1.255]	1.043 [0.655, 1.660]	1.075 [0.898, 1.288]	1.126 [0.965, 1.314]	1.156 [0.740, 1.807]	1.118 [0.947, 1.320]
Richer	1.010 [0.813, 1.254]	1.082 [0.706, 1.658]	0.788 [0.548, 1.134]	1.220* [1.046, 1.423]	1.243 [0.829, 1.864]	1.213* [1.022, 1.441]
Richest	1.028 [0.811, 1.305]	1.058 [0.687, 1.630]	1.165 [0.411, 3.298]	1.229* [1.023, 1.478]	1.321 [0.882, 1.977]	1.151 [0.868, 1.526]
Access to electronic media (no)	1	1	1	1	1	1
Yes	0.776** [0.657, 0.915]	1.315 [0.822, 2.102]	0.698*** [0.582, 0.835]	1.044 [0.943, 1.157]	0.990 [0.801, 1.223]	1.046 [0.930, 1.177]
Religion (Islam)	1	1	1	1	1	1
Other	1.090 [0.772, 1.539]	1.190 [0.766, 1.849]	0.987 [0.563, 1.729]	0.913 [0.828, 1.006]	0.898 [0.768, 1.049]	0.927 [0.818, 1.050]
Household head’s sex (male)	1	1	1	1	1	1
Female	0.836* [0.723, 0.967]	0.881 [0.729, 1.064]	0.780* [0.617, 0.986]	0.897* [0.822, 0.980]	0.926 [0.811, 1.059]	0.865* [0.768, 0.973]
History of abortion (no)	1	1	1	1	1	1
Yes	1.090 [0.923, 1.287]	1.076 [0.813, 1.425]	1.105 [0.897, 1.361]	0.808** [0.695, 0.938]	0.892 [0.729, 1.092]	0.712** [0.568, 0.892]
Observations	10,037	4421	5616	13,745	5804	7941
Pseudo *R*^2^	0.224	0.154	0.201	0.228	0.233	0.229

Exponentiated coefficients; 95% confidence intervals in brackets

*p < 0.05; **p < 0.01; ***p < 0.001

Having employment in a professional/technical/managerial position showed an inverse association with unmet need both in Gambia [Odds ratio = 0.843, 95% CI 0.730, 0.974] and Mozambique [Odds ratio = 0.886, 95% CI 0.786, 0.999]. The association between household wealth situation and unmet need was generally positive; however, there was no regular pattern in the association. For instance, the positive association was observed for richer [Odds ratio = 1.220, 95% CI 1.046, 1.423] and richest [Odds ratio = 1.229, 95% CI 1.023, 1.478] households in the pooled sample, but after stratification the association was significant only to rural women in the richer households [Odds ratio = 1.213, 95% CI 1.022, 1.441]. Having access to electronic media showed a negative effect on having unmet need [Odds ratio = 0.698, 95% CI 0.582, 0.835]. Living in male-headed households also showed a negative effect on unmet need both in Gambia [Odds ratio = 0.780, 95% CI 0.617, 0.986] and Mozambique [Odds ratio = 0.865, 95% CI 0.768, 0.973]. Rural women in Mozambique with history of abortion had lower odds of having unmet need for FP [Odds ratio = 0.712, 95% CI 0.568, 0.892].

## Discussion

In this country comparative study, the focus was on the prevalence and determinants of unmet need for FP among women in Gambia and Mozambique. Being located in two different regions in sub-Saharan Africa, these two countries share similar demographic and population health characteristics such as life expectancy, fertility rates, and maternal and child mortality rates. Our analysis indicates that unmet need for FP is similar in these two countries, with a slightly lower prevalence in Gambia (17.86%) than Mozambique (20.79%) and in comparison, with previous studies in Nigeria, 16.1% [20] and Ethiopia, 37.5% [21]. As estimated by the World Bank in 2016, the per capita health expenditure in Mozambique stands at $19.21 which is very close to that of Gambia, $20.93. However, meeting the reproductive health needs of Mozambique’s larger population presents a more sizeable task. In both of the countries, low coverage of FP remains a public health challenge because of the low use of contraception [22] and lack of adequate healthcare workers and healthcare infrastructure [23]. For this study, information on healthcare workforce and infrastructure—two important indicators for better coverage of FP services—was not available. However, region specific analysis showed that urban women in Mozambique had a noticeably lower prevalence of having unmet need for FP. Rural regions generally underperform in health-related indicators, and our analysis suggests that the urban–rural gap is also a matter of concern for Mozambique.

Several similarities were observed between these two countries in terms of the sociodemographic factors associated with unmet need as well as type of occupation and access to electronic media. In both countries, women having employment in white collar professions (e.g. technical/managerial) had lower likelihood of reporting unmet need of FP. In general, type of occupation reflects women’s socioeconomic status with professional jobs being more conducive to better health status and awareness [24]. This finding suggests that women without employment are at higher odds of having unmet need for FP, and therefore, deserve special attention from FP program designers. Although socioeconomic status is recognized as a key predictor of using FP services [25–28], our analysis did not find any significant association with wealth and educational status. Education plays a positive role on health knowledge and awareness which lead to better self-efficacy and practice [29, 30]. A study in the sub national region of Ethiopia also showed no association between education and unmet need for family planning [7]. Similarly, better financial situations act as an enabling factor for using health services, and therefore women living in wealthier households are believed to enjoy better access to contraception [31]. Despite these known positive roles, the insensitivity of education and wealth status results in this study can imply that the main causes of unmet need may lie outside the demand side factors and are rooted in supply side factors. Further studies are necessary to explore the mechanisms behind these irregularities.

Having access to electronic media showed an inverse association with unmet need for FP in Mozambique. This finding is line with several studies conducted in sub-Saharan African countries. The Nigeria Demographic and Health Survey (2013) revealed that women who had access to mass media messages had higher likelihood of using FP services [28]. Lack of access to mass media was found to be associated with higher level of unmet need for spacing and limiting births among women in Ethiopia [21]. Mass media platforms function as potential sources of health communication on various issues including sexual and reproductive health, thereby increasing exposure to information with the capacity to influence the knowledge and practice of seeking FP services. Concrete data on the content of the messages received through electronic media and whether they were relevant to FP was not available; this type of information could provide better context to inform our understanding of the association. FP communication through mass media [32] and social networking (including friends, family members, and media sources) [33] were shown to have beneficial effects on the use of FP. Based on these reports, it seems beneficial for family planning programs to utilize mass media as a knowledge mobilization tool for FP communication in Mozambique. For example, a recent study in Ghana revealed a high level of misconceptions about intrauterine devices among women that prevented use of the device for family planning [34]. Other countries experiencing low uptake of IUDs as contraception may benefit from mass media education dispelling myths and increasing successful FP approaches. Interestingly, no significant effect of media access was observed for Gambia; this requires further exploration.

Lastly, this study revealed women in female headed households and women who had experienced abortion were less likely to have unmet need for FP. A possible explanation for the first finding is that women in female headed households are more likely to be aware of the need for FP services, or enjoy a better FP-friendly environment than those who live in male headed households. For women who had a history of abortion, the likelihood of using FP is expected to be higher due the knowledge and awareness gained through their experience.

There are several limitations to report regarding the present analysis. Contraceptive use is a complex behaviour and can be influenced by a wide range of factors such as lack of awareness and knowledge, personal belief and attitude towards the technology, fear of side-effects, as well as inconvenience/unsuitability [35]. Individual behaviour itself is shaped and influenced by various sociocultural and environmental factors which are essential for a deeper understanding of the causes of non-use of FP services. As such, the subject matter is more qualitative in nature and requires in-depth investigation which is not possible through quantitative analysis. As this study was based on secondary data, authors had no control over the design of the study and were not able to choose variables in terms of their demonstrated association with unmet need for FP. Nonetheless, the findings provide valuable information for further qualitative research on this topic. Data collection took place in 2013 and 2011 for Gambia and Mozambique respectively, therefore, the prevalence estimates may not reflect the present scenario. Information on unmet need were self-reported, and thus remain subject to reporting bias/error. The surveys were cross-sectional, which means the outcome and explanatory variables were measured at the same time, and therefore cannot guarantee any causality of the associations.

## Conclusion

This study provides a comparative analysis of the situation of unmet need for FP in Gambia and Mozambique to determine prevalence and predictors of unmet need for contraception in the two low-income countries in sub-Saharan Africa with high fertility and maternal child mortality rates. Findings indicate that the prevalence in both countries are very similar, with about one-fifth of the women having unmet need of any type of FP (delaying or spacing). Several sociodemographic factors were found to be significantly associated with unmet need such as employment status, access to electronic media, sex of household head and history of abortion. The data may not reflect the current situation of unmet need since the surveys were conducted several years ago. Nonetheless, the present findings provide important insights for research and policy practices surrounding FP issues in these two countries. Further studies should be conducted to assess the level of awareness regarding FP and sociocultural barriers to the acceptance of this cost-effective technology.

## Supplementary Information

**Additional file 1**. Research methodology flowchart

## Data Availability

Data for this study were sourced from Demographic and Health surveys (DHS) and available here: http://dhsprogram.com/data/available-datasets.cfm.

## Abbreviations

DHS: Demographic and Health Surveys
MDGs: Millennium Development Goals
FP: Family Planning
SDGs: Sustainable Development Goals
