# Supplementary material for: Determinants of unmet need for family planning in Gambia & Mozambique: implications for women’s health
Source: BMC Womens Health. 2021 Mar 23;21:123. doi: 10.1186/s12905-021-01267-8 (PMC7989084; doi:10.1186/s12905-021-01267-8)
Supplement: Supplementary file 1 — Additional file 1. Research methodology flowchart [file 12905_2021_1267_MOESM1_ESM.pdf]

Research Methodology Flowchart

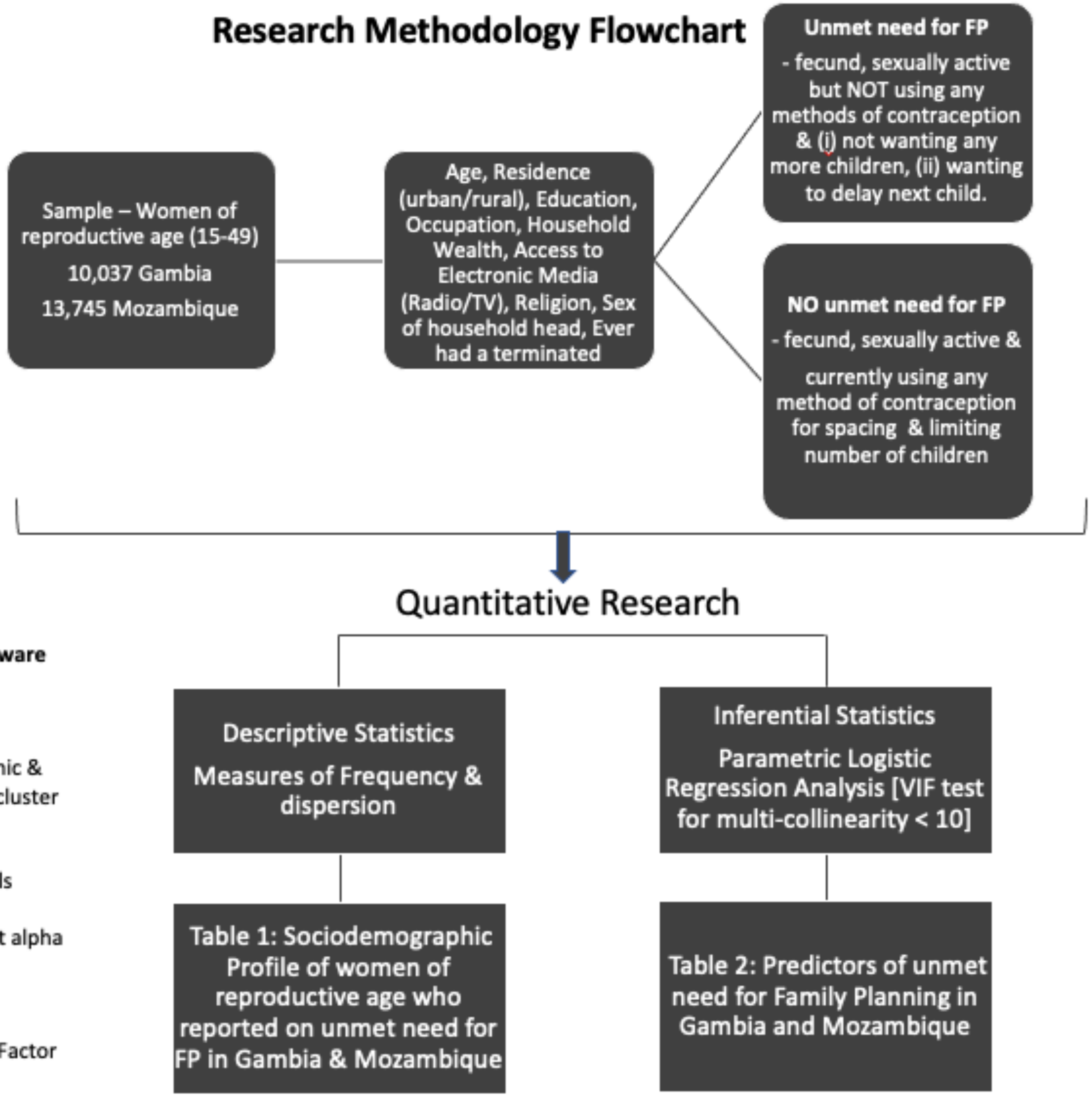

Statistical Analysis Software

STATA version 14.0
svy & *svyset* commands accounts for Demographic & Health Survey complex cluster sampling design

95% Confidence Intervals

Level of significant set at alpha value of 5%

FP = Family Planning
VIF = Variance Inflation Factor
